# Effect of repeated vitrification of human embryos on pregnancy and neonatal outcomes

**DOI:** 10.1186/s13048-024-01370-y

**Published:** 2024-02-24

**Authors:** Yan Huang, Yi Cheng, Min Zhang, Yiqing Chen, Rong Zhou, Dewei Lin, Xinyu Guo

**Affiliations:** 1 Reproductive Medicine Center, the General Hospital of Southern Theatre Command, Guangzhou, 510010 China; 2 The First Clinical Medical College, Southern Medical University, Guangzhou, China

**Keywords:** Repeated cryopreservation, Vitrification, Blastocyst, Pregnancy outcomes, Neonatal outcomes

## Abstract

**Background:**

Repeated cryopreservation of embryos should occasionally be considered when embryos were not suitable for transfer. The effect of re-cryopreservation on embryos remains contentious.

**Methods:**

This retrospective cohort study aimed to evaluate the pregnancy and neonatal outcomes of twice vitrificated blastocyst derived from once vitrified embryos. Total 410 vitrified-warmed blastocyst transfer cycles were divided into two groups according to the times of embryo vitrification: (1) vitrified blastocysts derived from fresh blastocysts (control group, *n* = 337); (2) twice vitrified blastocysts derived from once vitrified embryos (*n* = 73). The primary outcome was live birth rate. Multivariable logistic or linear regression analysis model was performed to describe the association between embryo cryopreservation times and clinical outcomes.

**Results:**

No difference was observed in female age at retrieval and transfer, infertility period, body mass index (BMI), infertility type, endometrial thickness, and embryo transfer numbers between the two groups. The pregnancy outcomes of embryos in repeated cryopreservation group were comparable to those of embryos in control group, including implantation rate, clinical pregnancy rate, and live birth rate. In multivariate logistic regression analysis, the cryopreservation times did not affect the outcomes of biochemical pregnancy, clinical pregnancy, and live birth. Moreover, there was no difference in gestational age, birthweight and sex ratio of singleton newborns between groups. After correcting several possible confounding variables, no significant association was observed between cryopreservation times and neonatal birthweight.

**Conclusion:**

In conclusion, pregnancy and neonatal outcomes achieved with twice vitrified blastocyst transfer were comparable to those achieved with vitrified blastocyst transfer in control group.

## Introduction

Embryo cryopreservation in human assistant reproduction technology (ART) has become a widespread reliable routine procedure. Various studies have showed the application of cryopreservation improved the cumulative live birth rate following a single ovarian stimulation cycle [1]. In addition, embryo cryopreservation technology reduced the risk of multiple pregnancies and ovarian hyperstimulation syndrome (OHSS) [2].

With the rapid development of ART and the increase in the clinical pregnancy rate and live birth rate, recent trends in IVF towards single embryo transfer for decreasing the risk of a multiple pregnancy. However, in many reproductive medicine centers, two or more embryos were frozen in one vitrification device for economic considerations. When frozen-thawed embryo transfer (FET), only one embryo was transferred, and the rest of embryos will be repeated cryopreserved. As a result, there may occasionally be surplus surviving embryos available for repeated vitrification, which can be transferred in the future. Likewise, in many centers, not all embryos are cultured to the blastocyst state to insure the available embryos for transfer. When numbers of cleavage embryos are cryopreserved, patients might choose bulk warming and extended culture to blastocyst for transfer. Therefore, surplus good-quality blastocysts remain to be re-cryopreserved after extended culture.

Dose repeated cryopreservation have negative effects on embryo quality and clinical outcomes? To our knowledge, there have been limited data on development of human embryos and clinical outcomes on repeated cryopreservation [3–11]. The pregnancy rates, miscarriage rate and live birth rate achieved with repeated vitrified blastocysts were comparable to those achieved with once vitrificated embryos [7]. And implantation rate, clinical pregnancy rate and miscarriage rates were comparable between twice cryopreserved group and control group with the vitrification method [5]. Wang et al. [10]. reported that the implantation rate, clinical pregnancy rate and live birth rate in the twice vitrification group were significantly lower. However, the number of transferred embryos was higher in once-cryopreservation group [10]. In another report, repeated vitrification showed high survival rates, similar clinical pregnancy rate and live birth rate independently of cryopreservation method and stages of embryonic development [9]. However, the live birth rate of repeated vitrified blastocysts derived from slow frozen-warmed day 3 embryos was significantly lower than that in the once vitrification group, implying the cryopreservation method might be associated with the clinical outcomes [11]. The transfer of twice frozen-thawed embryos with slow freeze technique showed lower pregnancy rate [3]. Due to the heterogeneity between these studies, the effect of repeated cryopreservation on human embryos remains controversial. Furthermore, few studies simultaneously focused on the embryonic development potential, clinical outcomes, and the neonatal outcomes of human embryos experiencing twice-vitrification [8, 10]. Therefore, to further clarify the effect of twice-vitrification on pregnancy and neonatal outcomes, we retrospectively analyzed the pregnancy and neonatal outcomes of re-cryopreserved blastocyst derived from vitrified-thawed blastocyst or cleavage embryos in the present study.

## Materials and methods

### Ethical approval and study design

The retrospective study was approved by the Medical Ethics Committee of General Hospital of Southern Theater Command (reference number: NZLLKZ2024018). We retrospectively reviewed the clinical data of frozen-thawed cycles from June 2017 to July 2021 at the Reproductive Medicine Centre of General Hospital of Southern Theater Command. Patients with endometriosis or preimplantation genetic testing were excluded. Female age at retrieval and transfer, duration of infertility, type of infertility, and endometrial thickness were preferentially matched. There were a total 410 vitrified-warmed blastocyst transfer cycles, divided into two groups based on the times of embryo vitrification: (1) vitrified blastocysts derived from fresh blastocysts (control group, *n* = 337); (2) twice vitrified blastocysts derived from once vitrified embryos (*n* = 73).

### Ovarian stimulation and embryo culture

Ovulation induction protocols include a gonadotrophin-releasing hormone (GnRH) antagonist protocol, or a short or long GnRH analogue suppression protocol combined with recombinant FSH. Oocyte cumulus complexes retrieval was performed 36 h after the administration of hCG and fertilized by conventional IVF or ICSI. Normal fertilized zygotes were cultured in G1 medium (Vitrolife, Sweden) to cleavage embryos for fresh embryo transfer, vitrification or blastocyst culture. The embryos transferred to G2 medium (Vitrolife, Sweden) were cultured to the blastocyst stage for fresh embryo transfer or vitrification. Garnder scoring system were used to evaluated blastocyst morphology based on the inner cell mass and trophectoderm grading [12].

### Vitrification and warming protocol

Vitrification of cleavage embryos and blastocysts was performed using a commercial kit (Kitazato company, Japan). Artificial shrinkage of the fully expanded blastocyst was performed before vitrification. Briefly, the embryos were equilibrated for 8–12 min in equilibration solution (12 min for blastocysts and 8 min for cleavage embryos), then placed in vitrification solution for 45–60 s. Finally, embryos were placed on the Cryotop strip (Kitazato company, Japan), and quickly immersed into liquid nitrogen. Warming of embryos were performed using a commercial kit (Kitazato company, Japan). Before the thawing, the thawing solution was preheated to 37 ℃ for 30 min. Then the Cryotop strip was quickly immersed into thawing solution. After 1 min, the embryos were removed into dilution solution for 3 min. Subsequently, the embryos were transferred into washing solution 1 and 2 for 5 min, respectively. Finally, the embryos were placed in G2 medium for culture or transfer.

### Artificial shrinkage of blastocyst

Artificial shrinkage was performed using the RI Saturn Laser System (England). The inner cell mass was positioned at a distance far from the shooting spot. One single laser shot (400 µs) was used to generate a small hole in one of the trophoblast cells.

### Blastocyte grade

Blastocyte grade was assessed according to the Gardner grading systems [12]. ICM quality was characterized by three grades: A, a tightly packed ICM with many cells; B, loosely grouped ICM with several cells, and C, very few cells and disorganized. TE quality was defined by a further three grades: A, denoting TE with many cells forming a cohesive epithelium; B, few cells forming a loose epithelium, and C, very few large cells. The blastocysts with high ICM/TE gradings (AA, AB, BA, and BB) were considered as high-grade, and other ICM/TE gradings (AC, CA, BC, CB, and CC) were considered as low-grade based on a precious report [13].

### Clinical parameters

The primary outcome was live birth rate per FET cycle. Prespecified secondary efficacy outcomes included biochemical pregnancy rate, implantation rate, clinical pregnancy rate, miscarriage rate, and neonatal outcomes. The biochemical pregnancy rate is calculated as the number of FET cycles resulting in a positive hCG level divided by the number of FET cycles. The implantation rate is calculated as the number of gestational sacs divided by the number of embryos transferred. The clinical pregnancy rate is calculated as the number of FET cycles resulting in at least one gestational sac with a heartbeat divided by the number of FET cycles. Miscarriage is defined as the pregnancy loss within 20 weeks. The miscarriage rate was calculated as the number of pregnancy loss cycles divided by the number of clinical pregnancy cycles. The live birth rate was calculated as the number of deliveries resulting in at least one live birth divided by the number of FET cycles. Neonatal outcomes include gestational age, preterm labor rate, birthweight, and gender. Preterm labor was defined as birth after week 20 and before week 37.

### Statistical analysis

The results were analyzed using SPSS version 27.0. Normality of data distribution was assessed by the Shapiro-Wilk test. Differences between once-vitrified group and twice-vitrified group were assessed through Student’s t-test for continuous data and Mann–Whitney U test for skews data. Chi-squared test was used to compare the differences of categorical data. For clinical outcomes and singleton birthweight, multivariate regression analysis was performed to assess these factors affecting outcomes. *P* < 0.05 was considered as statistically significant.

## Results

### Baseline clinical characteristics of the study cohort

In this retrospective analysis, 410 cycles were included: control group (*n* = 337) and re-vitrified group (*n* = 73). There was no difference in female age at retrieval and transfer, infertility period, BMI, infertility type, endometrial thickness, and embryo transfer numbers between the two groups (Table 1).

**Table 1:** Characteristics of included participants

	Control	Twice-vitrification	*P*
Cycles	337	73	
Female age at retrieval^%^ (y) Female age at transfer^%^ (y)	32.60 ± 4.07 33.32 ± 4.08	31.77 ± 4.18 34.46 ± 3.90	0.980 0.415
BMI^%^ (kg/m^2^) Duration of infertility^#^ (y) Type of infertility	22.15 ± 3.07 4 (2–5)	21.50 ± 2.61 4 (2–7)	0.141 0.186 0.916
Primary Secondary	187 150	41 32	
Endometrial thickness^#^ (mm) Blastocyst grade	10.3 (9.0-11.65)	10.8 (9.0–12.0)	0.345 0.904
High-grade Low-grade	400 25	85 5	
Average No. of embryos transferred^%^	1.26 ± 0.44	1.23 ± 0.43	0.500

BMI, Body mass index

^%^Student’s t-test. ^#^ Mann–Whitney U test.

### Clinical outcomes between the control and re-vitrified group

The blastocyst survival rate was 98.45% and 99.01% in the control group and re-vitrified group, respectively. No significant difference was found in the blastocyst survival rate after cryopreservation (*P* > 0.05).

The pregnancy outcomes were summarized in Table 2. The biochemical pregnancy rate (*P* = 0.625), implantation rate (*P* = 0.513), clinical pregnancy rate (*P* = 0.541), and live birth rate (*P* = 0.838) in the control group were comparable to those in the re-cryopreserved group. Likewise, there was no significant difference in the incidence of miscarriage or ectopic pregnancy between the two groups.

**Table 2:** Clinical outcomes of vitrified-warmed blastocyst transfer cycle

	Control	Twice-vitrification	*P*
Biochemical pregnancy rate^#^ (%) Implantation rate^#^ (%) Clinical pregnancy rate^#^ (%) Miscarriage rate^#^ (%) Live birth rate^#^ (%)	55.19 (186/337) 40.70 (173/425) 47.78 (161/337) 28.57 (46/161) 34.12 (115/337)	52.05 (38/73) 44.44 (40/90) 43.38 (32/73) 25 (8/32) 32.87 (24/73)	0.625 0.513 0.541 0.681 0.838
Signletons Twins	105 10	20 4	
Ectopic pregnancy rate^&^ (%)	3.10 (5/161)	6.25 (2/32)	0.725

^#^Pearson’s chi-squared test. ^&^ Fisher’s precision probability test.

### Exploration of factors associated with pregnancy outcomes using binary logistic regression models

Multivariate binary logistic regression was used to explore the effect of all related variables on pregnancy outcomes, including biochemical pregnancy rate, clinical pregnancy rate, and live birth rate. The variables included age at retrieval, BMI, endometrial thickness, embryos transferred numbers, and times of vitrification. As shown in Table 3, numbers of embryos transferred was significantly associated with biochemical pregnancy rate and clinical pregnancy rate. And endometrial thickness was significantly associated with biochemical pregnancy rate. Female age at retrieval was significantly associated with biochemical pregnancy rate and live birth rate. The number of vitrification times was not associated with the outcomes of biochemical pregnancy, clinical pregnancy, and live birth (*P* > 0.05 for all).

**Table 3:** Results of Logistic regression analysis of clinical outcomes

	Biochemical pregnancy rate				Clinical pregnancy rate			Live birth rate	
	OR (95%CI)	*P*			OR (95%CI)	*P*		OR (95%CI)	*P*
Female age at retrieval (y) BMI Endometrial thickness NO. of embryos transferred Embryo cryopreservation times	0.945 (0.896,0.997) 1.039 (0.969,1.115) 1.106 (1.007,1.215) 1.910 (1.163,3.139) 0.943(0.482, 1.848)		0.037^*^ 0.278 0.035^*^ 0.011^*^ 0.865		0.950(0.901,1.002) 1.024 (0.956,1.097) 1.086(0.989,1.193) 1.734 (1.076,2.795) 1.124 (0.581,2.174)	0.058 0.502 0.083 0.024^*^ 0.728		0.917 (0.861,0.976) 1.009 (0.931,1.092) 1.091 (0.979,1.216) 1.595(0.916,2.778) 0.000 (0.000)	0.006^*^ 0.831 0.115 0.099 0.998

BMI, Body mass index

Data are odds ratios (OR) with 95% confidence interval (CI).

^*^Difference is considered significant.

### Neonatal outcomes between the control group and re-vitrified group

The neonatal outcomes were summarized in Table 4. No significant difference in birthweight and gestational age was observed between the two groups. Likewise, preterm labor rate was 7.62% and 10.00% in control and twice-cryopreserved groups, respectively, with no significant difference. There were no differences in the percentage of boys born between the two groups (65.71% vs. 65.00%, *P* > 0.05).

**Table 4:** Neonatal outcomes of vitrified-warmed blastocyst transfer cycle

	Control	Twice-vitrification	*P*
cycles	105	20	
Gestational age^%^ (week)	38.27 ± 1.70	37.84 ± 1.91	0.345
Birthweight^%^ (g)	3223.61 ± 485.56	3353.12 ± 560.79	0.332
Preterm labor^&^	7.62 (8/105)	10.00 (2/20)	0.661
Gender^#^			0.951
Boy Girl	65.71 (69/105) 34.29 (36/105)	65.00 (13/20) 35.00 (7/20)	

^%^ Student’s t-test. ^&^ Fisher’s precision probability test. ^#^Pearson’s chi-squared test.

The adjustment variables such as female age at retrieval and transfer, BMI, gestational age and gender may affect the association between birthweight and times of embryo cryopreservation. Linear regression analysis model was established to calculate the associations between birthweight and embryo cryopreservation times, and it was corrected for the possible confounding variables. Embryo cryopreservation times was not related to the neonatal birthweight (Table 5). However, gestational age (*P* = 0.000) and gender (*P* = 0.001) were both independent predictors for birthweight.

**Table 5:** Results of linear regression analysis of singleton birthweight

Independents	Unstandardized coefficients			Standardized coefficients	t	*P*
	B	SE		Beta		
Constant	-4735.001	968.552			-4.889	0.000^*^
Female age at retrieval (y)	-42.696	32.844		-0.316	-1.300	0.196
Female age at transfer (y)	56.508	31.964		0.436	1.768	0.080
BMI (kg/m^2^) Embryo cryopreservation times	11.791 55.294	10.905 126.089		0.074 0.033	1.081 0.439	0.282 0.662
Gestational age, (week) Gender	205.564 -238.175	21.476 71.284		0.701 -0.228	9.572 -3.341	0.000^*^ 0.001^*^

BMI, Body mass index

Data are odds ratios (OR) with 95% confidence interval (CI).

^*^Difference is considered significant.

## Discussion

With significant advances in the field of IVF, the number of high-quality embryos has increased. Cryopreservation of embryos is a widespread reliable program during the ART clinical treatment. Currently, the clinical outcomes of FET are comparable to those of fresh embryos transfer cycles [14]. However, there are only limitation studies on the influence of repeated cryopreserved embryos on pregnancy and neonatal outcomes. In our study, the outcomes of repeated cryopreservation were comparable to those of once-cryopreserved embryos, including clinical pregnancy rate, live birth rate and neonatal outcomes.

There have been a serious of studies demonstrating the safety and advantages of FET compared with fresh embryo transfer. No significant difference was observed in live birth rate between fresh embryo transfer and FET among ovulatory women with infertility [15], but FET resulted in a lower risk of OHSS [9, 15]. The incidence of obstetrical and perinatal complications, congenital anomaly, and neonatal death are comparable [15]. As FET might provide optional transfer time for couples and a more physiologic uterine environment, women are more inclined to choose FET [16].

The developmental stage is associated with successful vitrification and the subsequent development after thawing. Taketsuru et.al [17] indicated that the tolerance of rat embryos to vitrification increases with embryonic development. In this study, we also assessed the blastocyst survival rate and pregnancy outcomes between the twice vitrified-thawed blastocyst (*n* = 11) or vitrified-thawed blastocyst after extended culture of vitrified-thawed cleavage embryos(*n* = 62). No significant difference was found in the blastocyst survival rate after cryopreservation between the two groups. And the biochemical pregnancy rate (54.54% (6/11) vs. 51.61% (32/62), *P* = 0.858), clinical pregnancy rate (45.45% (5/11) vs. 43.55% (27/62), *P* = 0.907), and live birth rate (36.36% (4/11) vs. 32.26% (20/62), *P* = 0.789) were comparable between the two groups. However, Zheng et. al [11] showed the live birth rate of repeated vitrified blastocysts derived from slow frozen-warmed day 3 embryos was significantly lower than that in the once vitrification group, implied the cryopreservation method might be associated with the clinical outcomes.

Currently, there are debated results regarding the effect of repeated cryopreservation on clinical outcomes. The implantation rate was increased in repeated vitrified blastocysts derived from slow frozen-warmed day 3 embryos [8], but were comparable to control group in another study [11]. Wang et al. [10]. reported that the implantation rate in the twice vitrification group were significantly lower. The effect of repeated cryopreservation on clinical pregnancy rate and miscarriage rates was also confused. Kumasako et al. [5] reported that clinical pregnancy rate and miscarriage rates were comparable between twice cryopreserved group and control group with the vitrification method, which was accordant with the report from Mizobe et al. [7]. However, Zheng et. al [11] and Wang et al. [10]. reported that the miscarriage rates was increased in the twice cryopreservation group. In our study, the clinical outcomes of re-vitrified blastocysts derived from vitrified-warmed day3 or day5 embryos were comparable to those achieved with control group.

In our study, there was no differences in terms of re-expansion status and survival between repeated vitrificated and once-vitrified blastocysts, which was consistent with precious studies [4–6, 8, 9, 11]. And the survival rate of twice vitrification from first vitrified-thawed 2PN embryos was comparable to that of once vitrification [5]. A serious of studies demonstrating the safety and advantages of vitrification compared with slow freezing. Slow freezing can cause cells to dehydrate at a slow rate and prevent intracellular ice formation, while vitrification enables hydrated living cells to be cooled to cryogenic temperature without ice formation by using an extra highspeed cooling rate and high concentration cryoprotectants. Therefore, cryoinjury after vitrification is milder than that after slow freezing [18]. Meanwhile, the loading devices could promote the cooling rate when the direct contacting between the embryos and liquid nitrogen. And embryo warming using a series of media with gradually decreasing osmotic pressure in an effort to reduce osmotic shock [19]. And assisted shrinkage is developed to reduce blastocyst volume prior to vitrification. Assisted shrinkage has been shown to improve the outcome of vitrification of blastocysts. Performing assisted hatching prior to blastocyst vitrification allows better permeation of the cryoprotectants and better blastocyst dehydration [20]. Meanwhile, assisted hatching promote the implantation rate as freezing induces hardening of the zona pellucida of the oocytes [20].

Currently, the risks of neonatal after FET remain debatable [21]. Many studies have also shown that FET is associated with the risks of pregnancy-induced hypertension, large for gestational age, preterm birth, and high birthweight infants [22]. Dose repeated cryopreservation have negative effects on neonatal outcomes? Only limited studies focused on the neonatal outcomes of embryos undergoing twice cryopreservation [7, 10, 11, 23]. Zheng et al [11]. compared the neonatal outcomes of repeated vitrified blastocysts derived from slow frozen-warmed cleavage embryos with once vitrified blastocysts and showed that the mean gestational age, birthweight and newborn anomalies were comparable between control and twice cryopreservation group. Likewise, Shen et.al [23] demonstrated that the risk of adverse neonatal outcomes between the twice vitrification group and once vitrification group were comparable. In our study, no significant differences were observed in gestational age, birthweight, and neonatal abnormality between control and twice vitrified embryos, which was consistent with precious study [7, 10, 11, 23].

Our study has limitations. First, this study was a reproductive study. Second, some meaningful variables, such as the chromosomal of transferred embryos and the cycles of FET were not included, which might cause a bias. As some infertility factors, such as PCOS, are association with embryo development [24] and clinical outcomes [25], blastocysts from different infertility factors have not been furtherly analyzed.

Taken together, this study showed that the pregnancy outcomes of re-vitrification were comparable to those achieved with control group. Also, there were no differences in the neonatal outcomes. The study could offer reproductive clinicians and embryologists more confidence when patients might choose bulk warming and extended culture to blastocyst for transfer. And the application of re-vitrified embryo transfer should be considered to prevent wasting embryos. A long-term multi-center follow-up study with more larger sample size is needed to reinforce our results and the safety of the re-vitrification for newborns.

## Data Availability

The datasets used and/or analyzed during the current study are available from the corresponding author on reasonable request.

## Abbreviations

BMI: Body mass index
ART: Assistant reproduction technology
OHSS: Ovarian hyperstimulation syndrome
FET: Frozen-thawed embryo transfer
GnRH: Gonadotrophin-releasing hormone
PCOS: Polycystic ovary syndrome
ICM: Inner cell mass
TE: Trophectoderm

## References

[1] Zhu Q, Chen Q, Wang L, Lu X, Lyu Q, Wang Y (2018). Live birth rates in the first complete IVF cycle among 20 687 women using a freeze-all strategy. Hum Reprod.

[2] Chen H, Zhang L, Meng L, Liang L, Zhang C (2022). Advantages of vitrification preservation in assisted reproduction and potential influences on imprinted genes. Clin Epigenetics.

[3] Farhi J, Elizur S, Yonish M, Seidman DS, Shulman A, Schiff E (2019). Assessment of a double freezing approach in the management of surplus embryos in IVF. Reprod Biomed Online.

[4] Kang SM, Lee SW, Yoon SH, Kim JC, Lim JH, Lee SG (2013). Comparison of clinical outcomes between single and double vitrified-warmed blastocyst embryo transfer according to the day of vitrification. J Assist Reprod Genet.

[5] Kumasako Y, Otsu E, Utsunomiya T, Araki Y (2009). The efficacy of the transfer of twice frozen-thawed embryos with the vitrification method. Fertil Steril.

[6] Li J, Xiong S, Zhao Y, Li C, Han W, Huang G. Effect of the re-vitrification of embryos at different stages on embryonic developmental potential. Front Endocrinol. 2021;12. 10.3389/fendo.2021.653310 PMC8317612 34335464

[7] Mizobe Y, Kuwatsuru Y, Kuroki Y, Fukumoto Y, Tokudome M, Moewaki H (2021). The effect of repeated cryopreservation and thawing using cryotip on the clinical outcomes of embryos. Reproductive Med Biology.

[8] Murakami M, Egashira A, Murakami K, Araki Y, Kuramoto T (2011). Perinatal outcome of twice-frozen-thawed embryo transfers: a clinical follow-up study. Fertil Steril.

[9] Stanger J, Wong J, Conceicao J, Yovich J (2012). Vitrification of human embryos previously cryostored by either slow freezing or vitrification results in high pregnancy rates. Reprod Biomed Online.

[10] Wang M, Jiang J, Xi Q, Li D, Ren X, Li Z (2021). Repeated cryopreservation process impairs embryo implantation potential but does not affect neonatal outcomes. Reprod Biomed Online.

[11] Zheng X, Chen Y, Yan J, Wu Y, Zhuang X, Lin S (2017). Effect of repeated cryopreservation on human embryo developmental potential. Reprod Biomed Online.

[12] Gardner DK, Schoolcraft WB (1999). Culture and transfer of human blastocysts. Curr Opin Obstet Gynecol.

[13] Shi W, Zhou H, Chen L, Xue X, Shi J (2022). Live birth rate following frozen-thawed blastocyst transfer is higher in high-grade day 6 blastocysts than in low-grade day 5 blastocysts. Front Endocrinol (Lausanne).

[14] Simón C, Gómez C, Cabanillas S, Vladimirov I, Castillón G, Giles J (2020). A 5-year multicentre randomized controlled trial comparing personalized, frozen and fresh blastocyst transfer in IVF. Reprod Biomed Online.

[15] Shi Y, Sun Y, Hao C, Zhang H, Wei D, Zhang Y (2018). Transfer of fresh versus frozen embryos in ovulatory women. N Engl J Med.

[16] Mackens S, Santos-Ribeiro S, van de Vijver A, Racca A, Van Landuyt L, Tournaye H (2017). Frozen embryo transfer: a review on the optimal endometrial preparation and timing. Hum Reprod.

[17] Taketsuru H, Kaneko T (2018). Tolerance to vitrification of rat embryos at various developmental stages. Cryobiology.

[18] Levron J, Leibovitz O, Brengauz M, Gitman H, Yerushalmi GM, Katorza E (2014). Cryopreservation of day 2–3 embryos by vitrification yields better outcome than slow freezing. Gynecol Endocrinol.

[19] Cho HJ, Son WY, Yoon SH, Lee SW, Lim JH (2002). An improved protocol for dilution of cryoprotectants from vitrified human blastocysts. Hum Reprod.

[20] Zech NH, Lejeune B, Zech H, Vanderzwalmen P (2005). Vitrification of hatching and hatched human blastocysts: effect of an opening in the zona pellucida before vitrification. Reprod Biomed Online.

[21] Zaat TR, Brink AJ, de Bruin JP, Goddijn M, Broekmans FJM, Cohlen BJ (2021). Increased obstetric and neonatal risks in artificial cycles for frozen embryo transfers?. Reprod Biomed Online.

[22] Singh B, Reschke L, Segars J, Baker VL (2020). Frozen-thawed embryo transfer: the potential importance of the corpus luteum in preventing obstetrical complications. Fertil Steril.

[23] Shen X, Ding M, Yan Y, Huang C, Wang S, Zhou J (2023). Perinatal outcomes of singletons following double vitrification-warming procedures: a retrospective study using propensity score analysis. BMC Pregnancy Childbirth.

[24] Palomba S, Daolio J, La Sala GB (2017). Oocyte competence in women with polycystic ovary syndrome. Trends Endocrinol Metab.

[25] Palomba S (2021). Is fertility reduced in ovulatory women with polycystic ovary syndrome? An opinion paper. Hum Reprod.

